# Selective Hydrogenation of 5-Hydroxymethylfurfural to 2,5-Dimethylfuran Over Popcorn-Like Nitrogen-Doped Carbon-Confined CuCo Bimetallic Catalyst

**DOI:** 10.3389/fchem.2022.882670

**Published:** 2022-04-12

**Authors:** Peng Hao, Jianliang Zuo, Wurong Tong, Jing Lin, Qiying Wang, Zili Liu

**Affiliations:** School of Chemistry and Chemical Engineering, Guangzhou University, Guangzhou, China

**Keywords:** 5-hydroxylmethylfurfural, CuCo bimetallic catalyst, selective hydrogenation, N-doped carbon nanotubes, 2,5-dimethylfuran

## Abstract

A new type of biomass-based liquid fuel, 2,5-dimethylfuran (DMF), has attracted significant attention owing to its unique physical properties and carbon neutrality. It can be obtained from the hydrogenation of 5-hydroxymethylfurfural (HMF), an important biomass platform compound. In this study, we developed a nitrogen-doped carbon-confined CuCo bimetallic catalyst with a popcorn-like structure for the selective hydrogenation of HMF with high efficiency and adequate stability. Under optimized conditions, 100% HMF conversion and 93.7% DMF selectivity were achieved. The structure of the catalyst was characterized using XRD, XPS, SEM, and TEM. It was observed that carbon spheres, which were covered by nitrogen-doped carbon nanotubes, uniformly formed, while metal particles were confined in the nitrogen-doped carbon nanotubes. The popcorn-like structure exhibited a larger surface area and provided more contact sites, while the confined metal particles were the main active sites. The synergistic effect between Cu and Co was beneficial for DMF selectivity.

## Introduction

With the increasing consumption of fossil fuels, the problems of resource depletion and the environmental pollution caused by fossil fuels have become increasingly serious. Therefore, there is an urgent need to develop sustainable and clean energy sources. Among them, biomass energy has attracted the attention of researchers as it can realize the carbon cycle and effectively reduce environmental pollution. Notably, 2,5-dimethylfuran (DMF) is considered a high-quality biomass-based liquid fuel owing to its unique physical properties (Tian et al., 2010; Alamillo et al., 2012; Wang H et al., 2019), such as high energy density, high boiling point, and low solvency in water (compared to first-generation bioethanol), and it can be produced by the selective hydrogenation of 5-hydroxymethylfurfural (HMF). HMF is an important biomass-based platform compound obtained from the hydrolysis of cellulose (Hu et al., 2008; Guo et al., 2020). However, because of its abundant reactive functional groups, side reactions and overhydrogenation easily occur during the hydrogenation of HMF to DMF (Zhang et al., 2017; Mishra et al., 2020; Wang et al., 2021). Developing highly efficient catalysts for the selective hydrogenation of HMF to DMF remains a challenge.

The current catalysts for the hydrogenation of HMF can be divided into two types: noble and non-noble metal catalysts. The most important feature of precious metals such as Pd, Pt, Ru, and Rh, is that they can achieve a high yield under mild conditions. Wang et al. (2018) obtained >90% DMF yields using Pt-Co bimetallic catalysts, while Zu et al. (2014) obtained 93.4% DMF yields using Ru/Co_3_O_4_ catalysts, and Zhang J et al. (2019) obtained 89.7% DMF yields using PdCl_2_ at room temperature. However, noble metal catalysts are limited by their scarcity and high cost. Therefore, there is an urgent need to develop low-cost, non-noble metal catalysts. Commonly used non-noble metals are Co, Cu, Ni, and Fe. Yang et al. (2019) obtained a 94.1% DMF yield using a synthetic Co/rGO catalyst at 200°C. Akmaz et al. (2019) prepared a Mn/Co bimetallic catalyst and obtained a DMF yield of 91.8%. Zhang Z et al. (2019) prepared a Co–CoO_x_ catalyst and achieved a DMF yield of 83.3% after 12 h at 170°C. Generally, copper and cobalt are favorable for hydrogenation of C=O, C–O bond, bimetallic catalysts are more active than monometallic catalysts (Guo et al., 2021; Zhao et al., 2022). Although non-noble metal catalysts reduce the cost, they also introduce the disadvantages of requiring harsh reaction conditions and easy deactivation. Thus, the development of an efficient and stable non-noble metal catalyst is crucial. In addition, nitrogen-doped carbon materials have been widely used as catalyst supporter which can anchor and stabilize metal nanoparticles and promote electron transfer to improve the performance of catalysts (Wang et al., 2020; Guo et al., 2021). Based on the aforementioned information, in this study, a nitrogen-doped carbon-confined copper-cobalt bimetallic catalyst was synthesized using a two-step solvothermal-reducing calcination method. It can be observed that the catalyst has a popcorn-like structure, and the surface is evenly covered by carbon nanotubes, which provides more surface area for the contact between the catalyst and the substrate. It has been used in the selective hydrogenation of HMF to DMF and has achieved adequate results. The main paragraph text follows directly on here.

## Experimental Section

### Chemicals

Co (NO_3_)_2_•6H_2_O, 2-methylimidazole, 5-hydroxymethylfurfural (97.0%), 5-methylfurfural, 2-butanol, urea, and melamine were procured from Shanghai Macklin Biochemical Co. Cu (NO_3_)_2_•3H_2_O, methanol, tetrahydrofuran (THF), and isopropanol (IPA) were procured from Guangzhou Chemical Reagent Factory. Glucose was procured from Tianjin Zhiyuan Chemical Reagent Co. Ltd.

### Preparation of the Catalyst

Copper-cobalt bimetallic catalysts were prepared using a solvothermal-reducing calcination method with 2-methylimidazole as the nitrogen sources and both 2-methylimidazole and glucose as the carbon sources. 3.69 g 2-methylimidazole, 0.5 g glucose, cobalt nitrate hexahydrate and copper nitrate trihydrate with different Cu/Co molar ratios (the amount of Co remains constant) were dissolved in 60 ml of methanol and stirred for 10 min to facilitate dissolution. This solution was then transferred to a 100 ml Teflon reactor and maintained at 120°C for 12 h. After cooling to room temperature, the solution was filtered five times with methanol and transferred to a vacuum drying oven, where it was allowed to stay overnight at 80°C. Subsequently, the obtained brown precursor powder was calcined in a tube furnace under a reducing H_2_ (5%)/N_2_ atmosphere at a rate of 2°C/min to 440°C for 8 h and then at a rate of 2°C/min to 900°C for 2 h to obtain the target Cu–Co bimetallic catalyst. The prepared catalyst was named xCuCo–IG (where x represents the molar ratio of copper to cobalt, I represents the nitrogen source 2-methylimidazole, G represents the carbon source glucose). The monometallic catalysts were named Cu-IG and Co-IG, respectively.

For comparison, catalysts with melamine or urea as the nitrogen source were prepared and named 2CuCo–MG and 2CuCo–UG, respectively. A catalyst without a nitrogen source (2CuCo–G) and a catalyst without glucose (2CuCo–I) were also prepared. The catalyst calcined in a N_2_ atmosphere was named 2CuCo–IG (N_2_).

### Characterization

The X-ray diffraction (XRD) patterns of the powder samples were recorded using a BRUKER D8 ADVANCE diffractometer. X-ray photoelectron spectroscopy (XPS) data was measured using a Thermo ESCALAB 250Xi spectrometer. The specific surface area and porosity of the samples were obtained using a Micrometrics ASAP2460. The morphology of each sample was investigated using field emission scanning electron microscopy (SEM, SU8020), and the element mapping was performed using energy dispersive spectrometer (EDS). Transmission electron microscopy (TEM) and high-resolution transmission electron microscopy (HRTEM) images were obtained using a FEI Tecnai G2 F30. The Raman spectral profile was obtained using a Renishaw inVia at an excitation wavelength of 532 nm.

### Test of Catalytic Activity

The catalytic performance in the hydrogenation of HMF was investigated in a stainless-steel autoclave. First, 0.2 g of the substrate (HMF), 0.02 g of the catalyst, and 0.05 g of toluene were dissolved in 30 ml of 2-butanol and subsequently poured into the reactor. After installation, hydrogen was purged at least eight times to remove air and charged with H_2_ at the corresponding pressure; the agitation speed was modulated to 400 rpm, followed by an increase in the temperature to the target temperature of 180°C. After the reaction was completed and cooled to room temperature, the reaction products were analyzed using the internal standard curve method to determine their conversion and selectivity with an Agilent 6820 gas chromatograph. The calculations for the conversion of HMF and DMF selectivity were performed as follows:

$$\text{Conversion}\left(\text{HMF}\right)=\left(1-\frac{n_{t}}{n_{0}}\right)\times 100\text{\%},$$

$$\text{Selectivity}=\frac{n_{i}}{\text{Conv}.\text{HMF}}\times 100\text{\%},$$

where n_t_ represents the molar amount of HMF after the reaction, n_0_ represents the molar amount of initial HMF, and n_i_ represents the molar amount of the product after the reaction.

## Results and Discussion

### Structural Analysis

**FIGURE 1:**
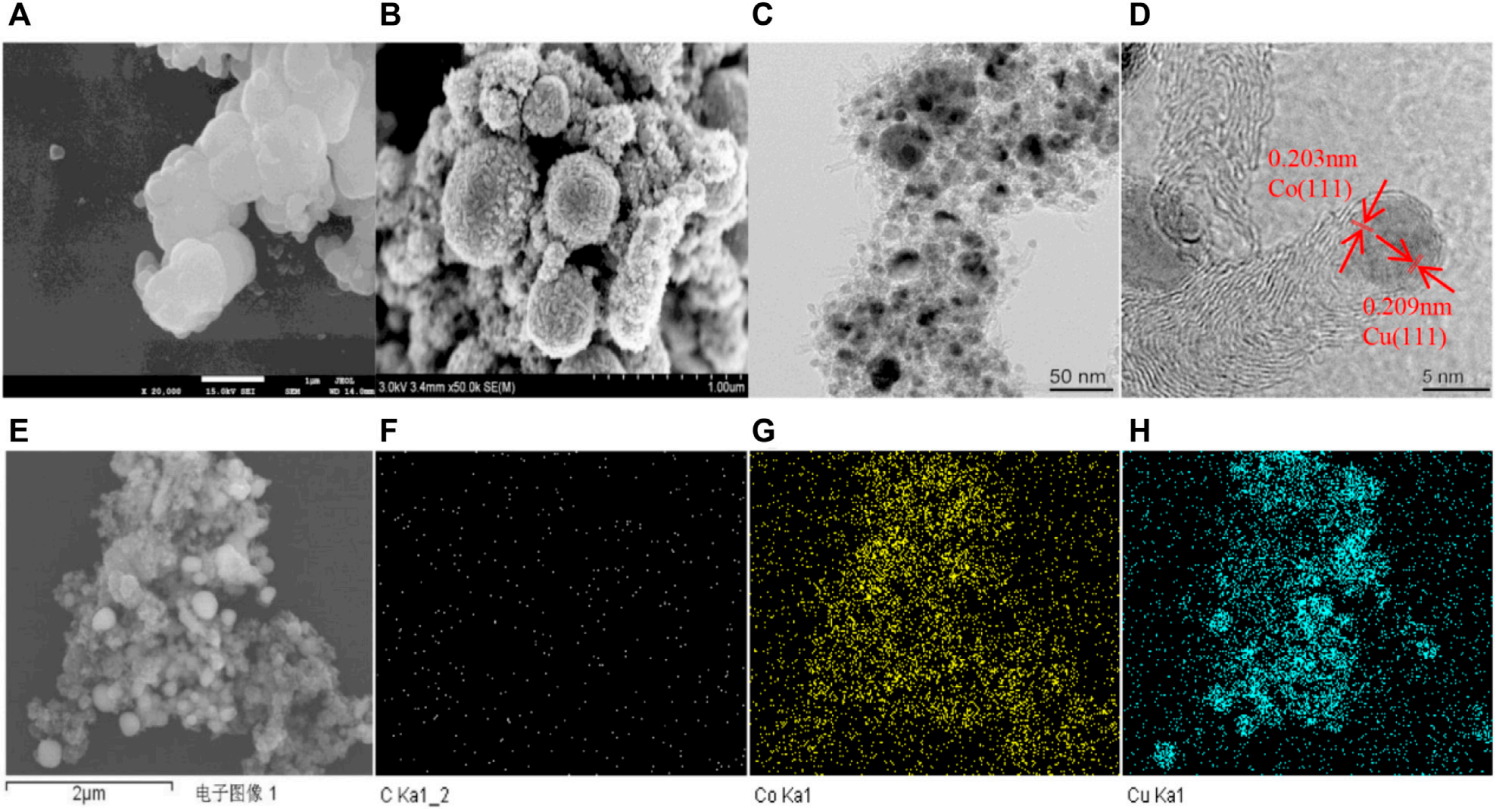
**(A)** SEM profiles of the precursor, **(B)** 2CuCo–IG obtained after the calcination of H_2_ (5%)/N_2_; **(C,D)** TEM and HRTEM profiles of 2CuCo-IG; **(E–H)** the SEM and elemental images of the catalyst 2CuCo–IG.

First, the 2CuCo–IG catalyst precursor with a Cu/Co molar ratio of 2:1 was synthesized using a one-pot solvothermal reaction of metal salts, glucose (as the carbon source), and 2-methylimidazolem (as the nitrogen source and carbon source). Precursors mainly showed spherical structures with non-smooth surfaces (Figure 1A). Soon after the calcination under a H_2_ (5%)/N_2_ mixture atmosphere, the catalyst exhibited a popcorn-like structure with carbon balls uniformly covered by carbon nanotubes (Figure 1B). TEM and HRTEM images (Figures 1C,D) indicated that carbon balls were covered by carbon nanotubes, and Cu–Co metal particles were confined to the tips of the nanotubes with an average size of 6–8 nm. Figure 1D shows a lattice fringe of 0.209 nm attributable to the plane of Cu (111) (Liu J et al., 2020; Viar et al., 2020), and a lattice distance of 0.203 nm attributable to the plane of metallic Co (111) (Ma et al., 2020). In addition, the SEM and elemental images of Cu, Co, C in the 2CuCo–IG catalyst showed that copper and cobalt were uniformly dispersed throughout the catalyst (Figures 1E–H).

To investigate the reason for the formation of carbon nanotubes, we prepared catalysts such as 2CuCo–G (no nitrogen source added), 2CuCo–UG (nitrogen source replaced by urea), 2CuCo–MG (nitrogen source replaced by melamine), and 2CuCo–I (no glucose carbon source added). As shown in Supplementary Figures S2A–D, it can be observed that when the nitrogen source was changed, the catalyst structure also changes and no longer develops a popcorn-like structure. When no glucose carbon source was added, no spherical support structure was formed, but the carbon nanotubes still appeared (Supplementary Figure S2D), which indicates that carbon nanotubes are most likely formed during the calcination of 2-methylimidazole. The effect of the calcination atmosphere on the morphology of the catalyst was also investigated (Supplementary Figure S1). The carbon nanotubes on the catalyst calcined under a N_2_ atmosphere are fewer and significantly finer than those formed under a mixed atmosphere. This means that both the formation and morphologies of carbon nanotubes are highly dependent on the nitrogen source and the calcination atmosphere.

**FIGURE 2:**
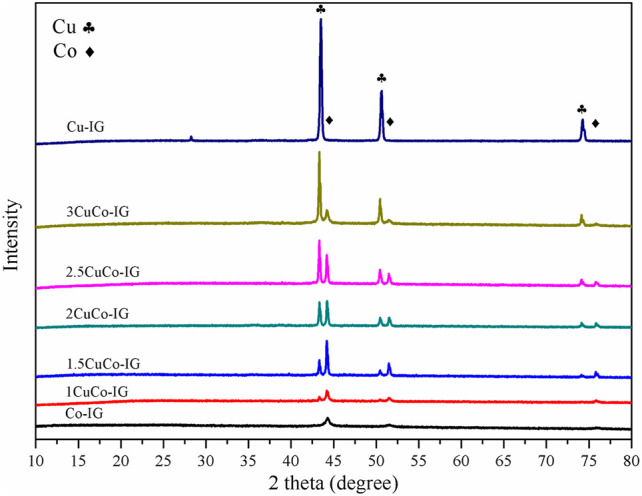
XRD patterns of the copper–cobalt bimetallic catalysts with different Cu/Co ratios.

A series of copper-cobalt bimetallic catalysts was prepared with different Cu/Co ratios, and their XRD patterns are shown in Figure 2. The diffraction peaks at 43.3°, 50.4°, and 74.1° are attributed to metallic copper (PDF #89-4307) (Chen et al., 2020; Rao et al., 2020; Xu et al., 2021), and those at 44.2°, 51.5°, and 75.9° are attributed to metallic cobalt (PDF #89-2838) (Chen et al., 2017; Solanki and Rode, 2019). It can be observed that mono metal catalyst Cu–IG contains only metallic copper diffraction peak, while Co–IG catalyst contains only metallic cobalt diffraction peak. The CuCo bimetallic catalyst contains both metallic copper and metallic cobalt diffraction peaks, with an increase in the ratio of copper to cobalt, the diffraction peaks of both elements exhibited different variation trends. The diffraction peaks of metallic copper continuously increased as the proportion of metallic copper increased. The diffraction peaks of metallic cobalt also increased initially. However, when this ratio was exceeded 2.5, the diffraction peaks of metallic cobalt decreased. This shows that a suitable ratio of copper to cobalt can improve the crystal structure of metallic cobalt.

The N_2_ adsorption/desorption curves of the catalysts are shown in Supplementary Figure S3. The existence of hysteresis loops indicated that the prepared catalysts were typical mesoporous materials. The specific surface area and pore size data are listed in Supplementary Table S1. Among the catalysts with different Cu/Co ratios, the 2CuCo–IG catalyst had the largest specific surface area, and the large pore volume and the adequate pore diameter improved contact, significantly improving the utilization of the catalyst.

**FIGURE 3:**
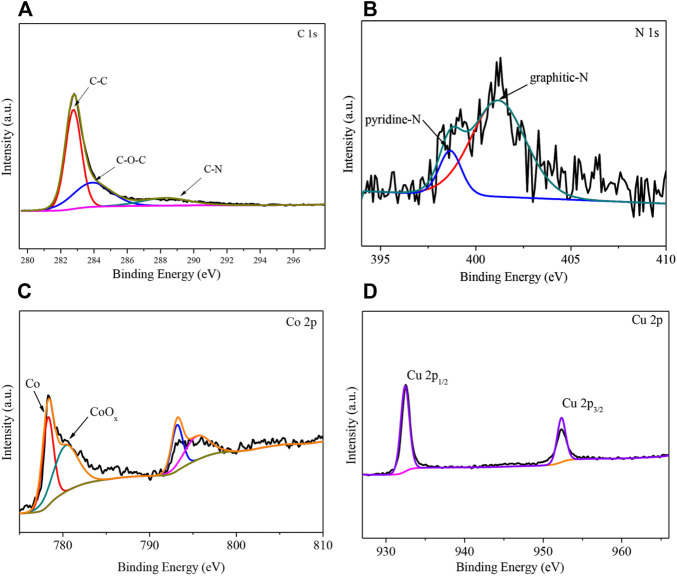
XPS spectra of the catalyst 2CuCo–IG. **(A)** High-resolution spectra of C 1s, **(B)** N 1s, **(C)** Co 2p, and **(D)** Cu 2p.

The XPS profiles of the 2CuCo–IG catalyst are shown in Figure 3. Two peaks are visible at 284.8 and 286.0 eV in the high-resolution XPS spectrum of C 1s mainly corresponding to C–C and C–O–C (Xia et al., 2016; Rao et al., 2021). The peak near 289.0 eV is attributed to C–N in the catalyst (Figure 3A) (Liu L et al., 2020; Zhu K et al., 2020). Two peaks were visible at 398.6 and 401.2 eV in the high-resolution XPS spectrum of N 1s corresponding to pyridine N and graphite N (Figure 3B) (Hu et al., 2019). The elemental nitrogen content was determined to be 2.5% using XPS, including 15.0% pyridine nitrogen and 85.0% graphite nitrogen. The high-resolution XPS spectrum of Co 2p shows the characteristic peak of Co^0^ at 778.3 eV (Liu M et al., 2020), while the peak at 780.3 eV corresponds to CoO_x_ (Figure 3C) (Zhu J et al., 2020). The diffraction peaks of Cu 2p1/2 and Cu 2p3/2 in the high-resolution XPS spectrum of Cu 2p were present at 952.3 and 932.3 eV; this indicates that the valence state of Cu is in the metallic phase (Figure 3D) (Kang et al., 2016), which is consistent with XRD results. The two peaks at 1350 and 1600 cm^−1^ in the Raman spectrum (Supplementary Figure S4) are the D and G bands, respectively (ID/IG = 0.9), indicating a relatively high degree of graphitization of carbon. It should be noted that high contents of graphitic carbon are conducive to the catalytic hydrogenation reaction (Gong et al., 2019).

### Hydrogenolysis of 5-Hydroxymethylfurfural to 2,5-Dimethylfuran

**FIGURE 4:**
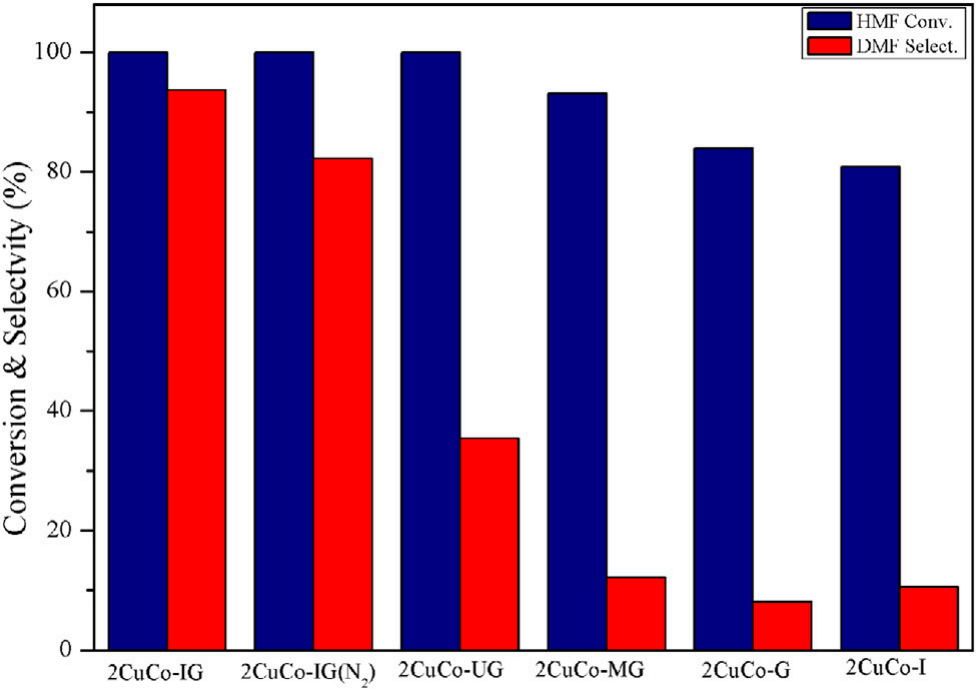
Effect of the different carbon/nitrogen sources and calcination atmospheres on catalyst performance. Reaction conditions: 0.2 g of HMF, 0.04 g of the catalyst, 30 ml of 2-Butanol, 2 MPa H_2_, 4 h, and an agitation speed of 400 rpm.

The effects of the different carbon/nitrogen sources and calcination atmospheres on the catalyst activity were also investigated (Figure 4). Both the catalyst without the addition of a glucose carbon source (2CuCo–I) and that without the addition of 2-methylimidazole as a nitrogen source (2CuCo–G) exhibited very low DMF selectivity. When the nitrogen source was changed to urea or melamine, the selectivity of DMF was only 35.4 and 12.2%, respectively. This implies that both carbon and nitrogen sources are indispensable, and that the type of nitrogen source also has a significant impact on DMF selectivity. The 2CuCo–IG (N_2_) calcined in a N_2_ atmosphere showed 82.3% DMF selectivity which is lower than that of the 2CuCo–IG calcined in a H_2_ (5%)/N_2_ atmosphere. This may be because the reducing atmosphere is beneficial for formation and dispersion of metallic CuCo particles. As showed in Supplementary Figure S1, more nitrogen doped carbon nanotubes in which CuCo bimetal particles were confined were formed in the 2CuCo–IG catalyst because of lower depletion of carbon under reducing atmosphere than inert atmosphere. The calcination atmosphere is also benefit for the thorough reduction of Cu although the carbon also can partially reduction of Cu. On other hand, The ratio of Co^0^/CoO_x_ in the 2CuCo–IG is higher than that in 2CuCo–IG (N_2_) although both of 2CuCo–IG and 2CuCo–IG (N_2_) catalyst contain metallic Co and oxidation state Co (Figure 3 and Supplementary Figure S5).

**FIGURE 5:**
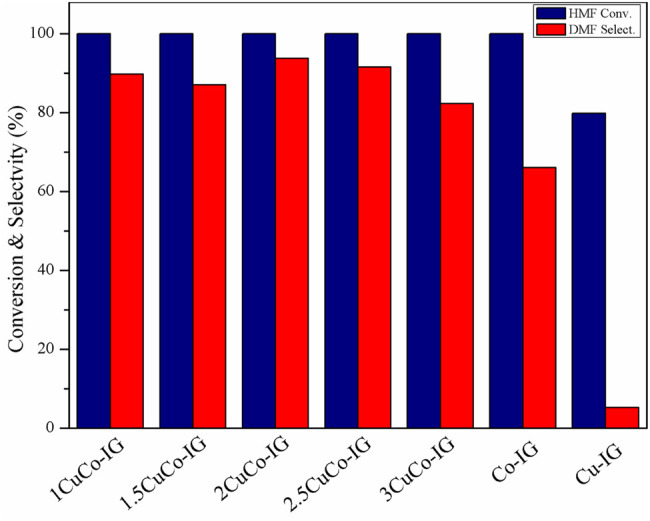
Effects of different copper-cobalt ratios on HMF conversion and the DMF yield. Reaction conditions: 0.2 g of HMF, 0.04 g of the catalyst, 30 ml of 2-Butanol, 2 MPa H_2_, 4 h, and an agitation speed of 400 rpm.

The effects of the different Cu/Co ratios on the catalyst activity are shown in Figure 5. HMF conversion was 79.8% and DMF selectivity was only 5.3% when a mono-copper metal was used as the catalyst, whereas 100% HMF conversion and 66.0% DMF selectivity were achieved when a mono-cobalt metal was used as the catalyst. All the CuCo bimetallic catalyst showed higher conversion and DMF selectivity than monometallic catalysts. The best proportion was obtained when the copper-to-cobalt ratio was 2, and a 93.7% DMF yield was achieved. By comparison, we speculated that there will be a synergistic effect between Cu and Co which played an important role in improving the catalytic performance.

**FIGURE 6:**
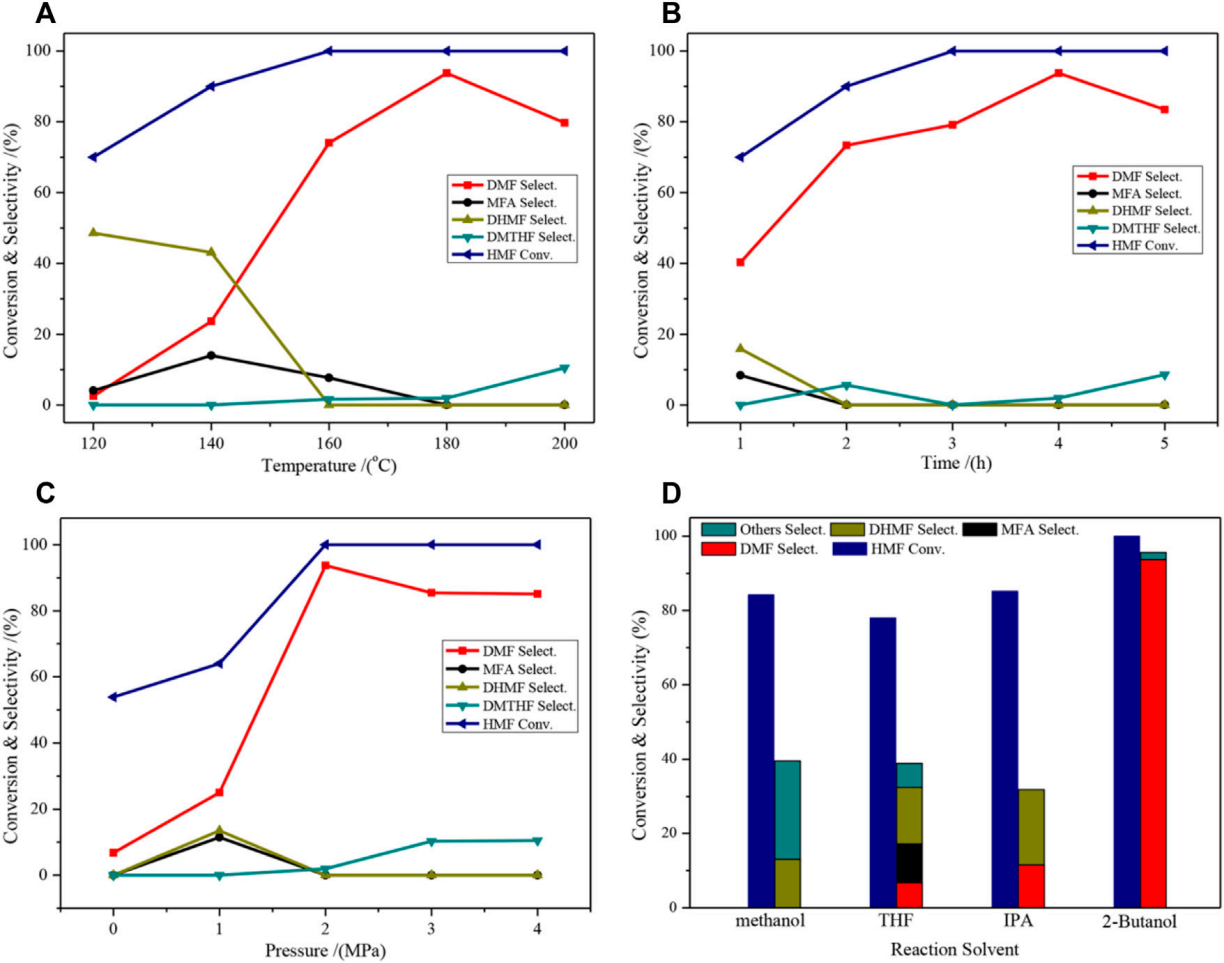
Catalytic performance of HMF hydrogenolysis and distribution of products on the 2CuCo–IG catalyst over different reaction conditions. **(A)** 4 h, 2.0 MPa H_2_, 30 ml of 2-Butanol; **(B)** 180°C, 2.0 MPa H_2_, 30 ml of 2-Butanol; **(C)** 4 h, 180°C, 30 ml of 2-Butanol; **(D)** 4 h, 180°C, 2.0 MPa H_2_, 30 ml of 2-Butanol.

The effects of the different reaction conditions on the catalytic activity of the selective hydrogenation of HMF to DMF were investigated (Figure 6). The selectivity of DMF increased when the temperature gradually increased, and the highest selectivity (93.7%) was obtained at 180°C. Further increasing the temperature will result in the deep hydrogenation of DMF in the C=C bond of the furan ring to form 2,5-Dimethyloxolane (DMTHF), resulting in a decrease in DMF selectivity (Figure 6A). The effect of the reaction time is comparable to that of the temperature (Figure 6B), and it shows the highest selectivity at 4 h. For the effect of the pressure factor, DMF selectivity rapidly increased when the pressure increased from 1 to 2 MPa; however, excessive pressure can also lead to overhydrogenation (Figure 6C). The effect of the reaction solvent on the activity is shown in Figure 6D. The use of different solvents caused dramatic changes in the activity level, which indicated that the solvent was present in the reaction. The effect of the solvent will be discussed in the following section. Overall, the catalyst showed the best performance when 2-butanol was used as the solvent.

**FIGURE 7:**
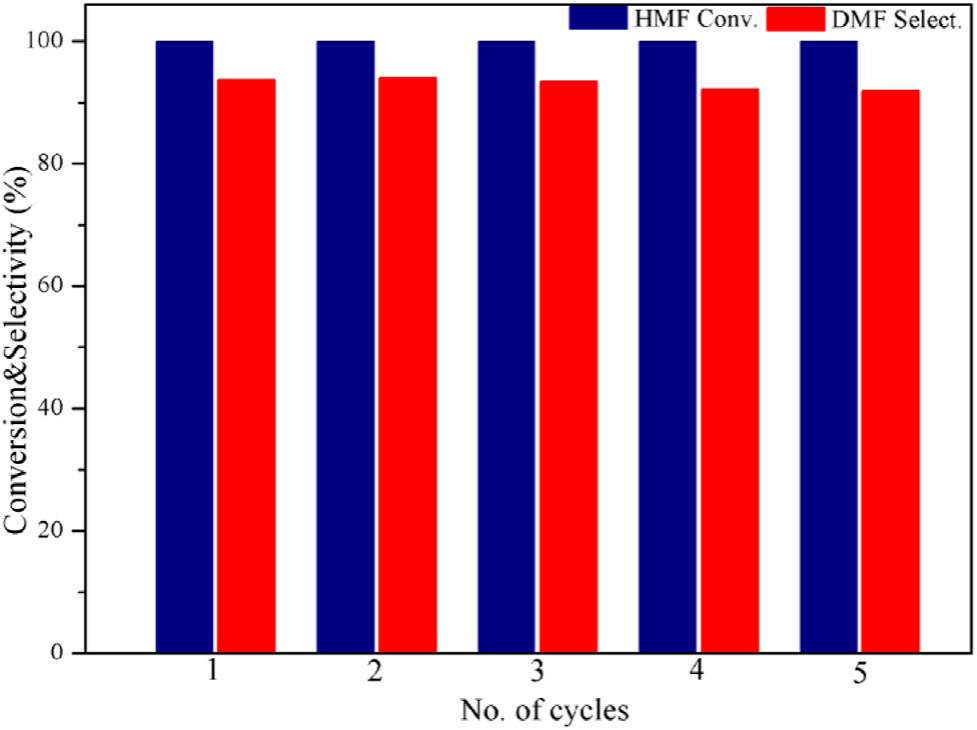
Catalyst cycling performance test. Reaction conditions: 0.2 g of HMF, 0.04 g of the catalyst, 30 ml of 2-Butanol, 2 MPa H_2_, 4 h, and an agitation speed of 400 rpm.

The cycling performance of the catalyst is shown in Figure 7. Since the catalyst itself is magnetic, it can be recovered easily after the reaction. After five cycles, no distinct decrease was observed in either HMF conversion or DMF selectivity. This indicates that the catalyst is highly stable and adequately reusable.

### Reaction Pathway

**FIGURE 8:**
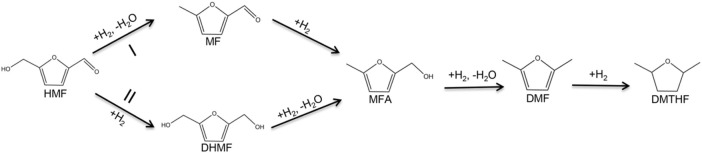
Reaction pathways of the selective hydrogenation of HMF to DMF.

The hydrogenation of HMF to DMF is divided into two main pathways: routes I and II (Figure 8) (Nakagawa et al., 2013; Qian et al., 2015; Wang Q et al., 2019). Route I is the hydrogenation and dehydration of HMF to produce MF, by further hydrogenation to produce MFA and finally DMF. Route II is the hydrogenation of HMF to produce DHMF, followed by hydrogenation and dehydration to produce MFA and finally DMF. DMF could also be transformed into DMTHF by the C=C hydrogenation in the furan ring. The results of the time optimization experiment (Figure 6B) showed that the intermediate product that appeared at 1 h was DHMF, indicating that the reaction process was route II.

To further study the effect of the solvent on product selectivity and the role of Cu/Co at each step of the hydrogenation reaction, a series of hydrogenation reactions was designed using MF with an aldehyde group and a furan ring and MFA with a hydroxyl group and a furan ring as substrates (Tables 1, 2). When methanol was used as the solvent, the selectivity of DMF was lower than that when 2-butanol was used, in both the hydrogenation of MF and MFA. Furthermore, when methanol was used, more etherification occurred, and more condensation products were formed. This may be due to the steric effects of 2-butanol that suppressed etherification and condensation reactions and resulted in the target product DMF.

**TABLE 1:** Effects of the different reaction conditions on MF conversion and products.

Catalyst	Gas	Solvent	MF Conv.(%)	MFA Select.(%)	DMF Select.(%)	Others Select.(%)
2CuCo–IG	H_2_	2-Butanol	78.0	54.8	20.2	0
2CuCo–IG	H_2_	Methanol	97.0	17.0	10.0	36.6
2CuCo–IG	N_2_	2-Butanol	47.3	33.4	8.5	0
1CuCo–IG	H_2_	2-Butanol	70.0	31.0	33.6	0

Reaction conditions: 0.2 g of MF, 0.02 g of the catalyst, 140°C, 2 h, 2 MPa corresponding gas, 30 ml of the corresponding solvent, and an agitation speed of 400 rpm.

**TABLE 2:** Effects of the different reaction conditions on MFA conversion and products.

Catalyst	Gas	Solvent	MFA Conv. (%)	DMF Select (%)	DMTHF Select (%)	Others Select (%)
2CuCo–IG	H_2_	2-Butanol	72.5	56.7	10.9	0
2CuCo–IG	H_2_	Methanol	60.4	6	0	53.9
2CuCo–IG	N_2_	2-Butanol	45.8	35.3	5.0	0
1CuCo–IG	H_2_	2-Butanol	82.3	64.7	17.3	0

Reaction conditions: 0.2 g of MFA, 0.02 g of the catalyst, 140°C, 2 h, 2 MPa corresponding gas, 30 ml of the corresponding solvent, and an agitation speed of 400 rpm.

The effects of the different Cu/Co ratios on the reaction process were investigated (Tables 1, 2). A series of hydrogenation reactions was designed using MF with an aldehyde group and a furan ring and MFA with a hydroxyl group and a furan ring as substrates. In the one and four lines of Table 1, it was observed that the higher the copper content, the higher the MF conversion and MFA selectivity in the hydrogenation reaction of MF, which indicates that metallic copper is active for the hydrogenation of C=O bonds. Meanwhile, the higher the cobalt content, the higher the MFA conversion and DMF selectivity in the hydrogenation reaction of MFA, which indicates that Co/CoO_x_ is more active for the hydrogenation of C–O bonds. The synergistic effect of copper and cobalt promotes the whole hydrogenation process.

## Conclusion

In summary, a popcorn-like nitrogen-doped carbon-confined CuCo bimetallic catalyst was prepared using a two-step solvothermal-reducing calcination method. The 2CuCo–IG catalyst performed well in the HMF selective hydrogenation to DMF with an HMF conversion of 100% and a DMF yield of 93.7%. The popcorn-like structure provided more active sites and electrons, and the confinement effect of nitrogen-doped carbon nanotubes and the synergistic effect of copper and cobalt were the main reasons for the high catalytic efficiency. The 2-butanol solvent not only provided hydrogen but also reduced the unwanted reactions of etherification and condensation using steric effects during the reaction. Meanwhile, the catalyst exhibited adequate recycling performance; thus, it can be reused.

## Data Availability

The original contributions presented in the study are included in the article/Supplementary Material, further inquiries can be directed to the corresponding authors.
