# Supplementary material for: Selective Hydrogenation of 5-Hydroxymethylfurfural to 2,5-Dimethylfuran Over Popcorn-Like Nitrogen-Doped Carbon-Confined CuCo Bimetallic Catalyst
Source: Front Chem. 2022 Apr 12;10:882670. doi: 10.3389/fchem.2022.882670 (PMC9039160; doi:10.3389/fchem.2022.882670)
Supplement: Supplementary file 1 [file DataSheet1.docx]

Selective Hydrogenation of 5-Hydroxymethylfurfural to 2,5-Dimethylfuran over Popcorn-Like Nitrogen-Doped Carbon-Confined CuCo Bimetallic Catalyst Supplementary Material


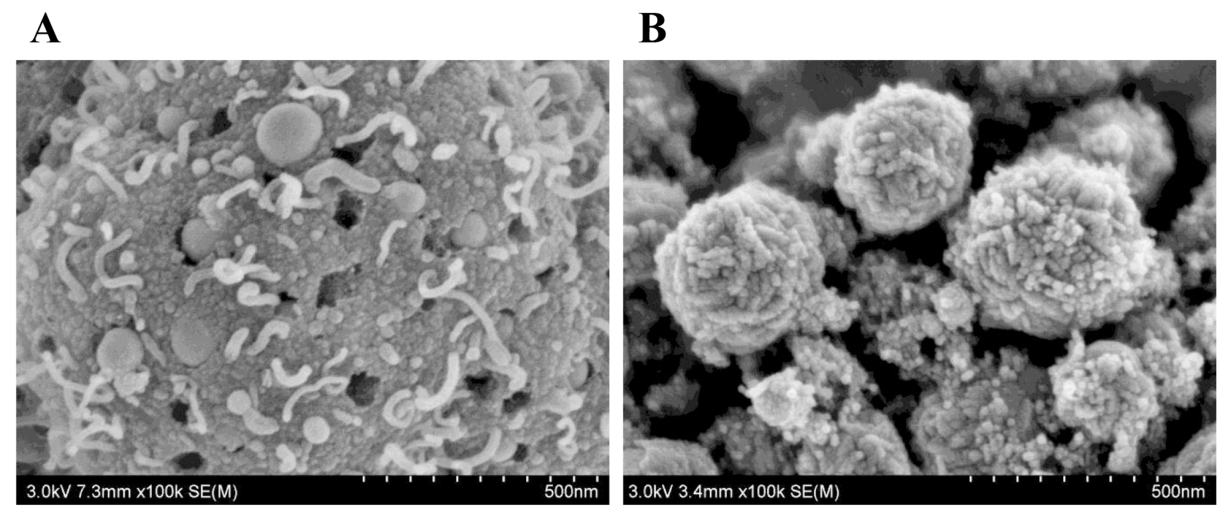


Figure S1 (A) SEM analysis of 2CuCo-IG(N_2_) catalyst ; (B) SEM analysis of 2CuCo-IG catalyst.


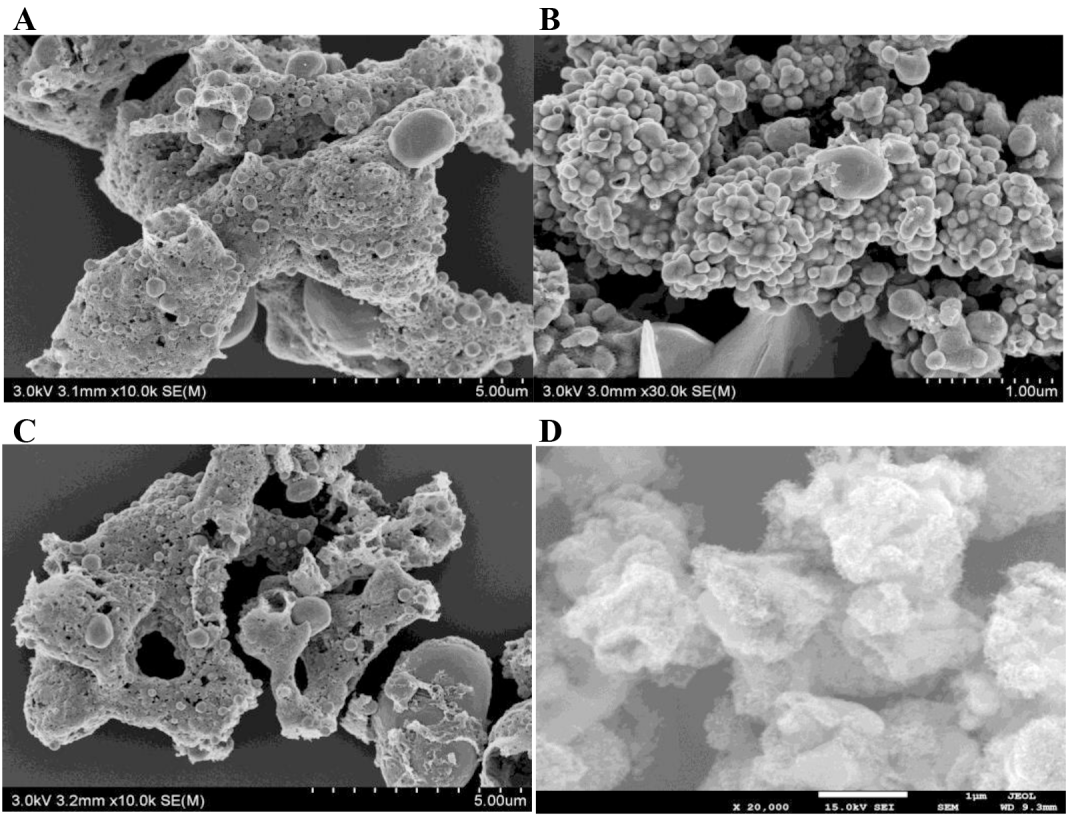


Figure S2 SEM analysis of the effect of different nitrogen/carbon source on catalyst structure. (A) No nitrogen source added: 2CuCo-G; (B) Nitrogen source is urea: 2CuCo-UG; (C) Nitrogen source is melamine: 2CuCo-MG; (D) No carbon source added: 2CuCo-I.





**Figure S3** N_2_ adsoption/desorption isotherms of CuCo bimetallic catalyst.





Figure S4 Raman spectra of 2CuCo-IG.


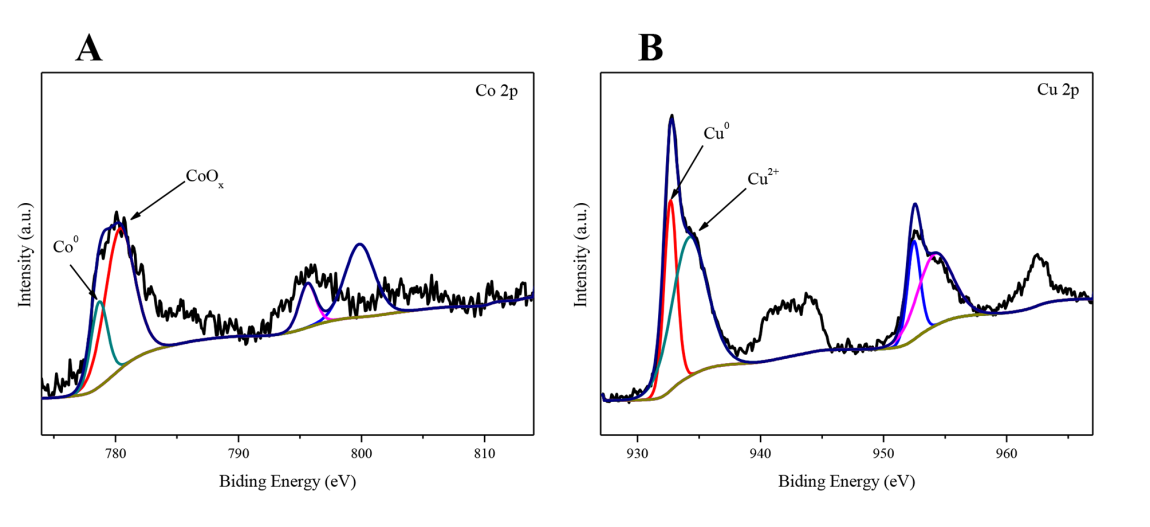


**Figure S5** XPS profiles of 2CuCo-IG(N_2_). (A) Co 2p of 2CuCo-IG(N_2_); (B) Cu 2p of 2CuCo-IG(N_2_).

Table S1 The specific surface area and porosity of CuCo bimetallic catalyst

| Catalyst | BET surface area  (m^2^·g^-1^) | Pore Volumes  (cm^3^·g^-1^) | Pore size  (nm) |
| --- | --- | --- | --- |
| 3CuCo-IG | 63.2 | 0.09 | 112.3 |
| 2CuCo-IG | 86.2 | 0.13 | 121.1 |
| 1CuCo-IG | 49.2 | 0.14 | 142.2 |
